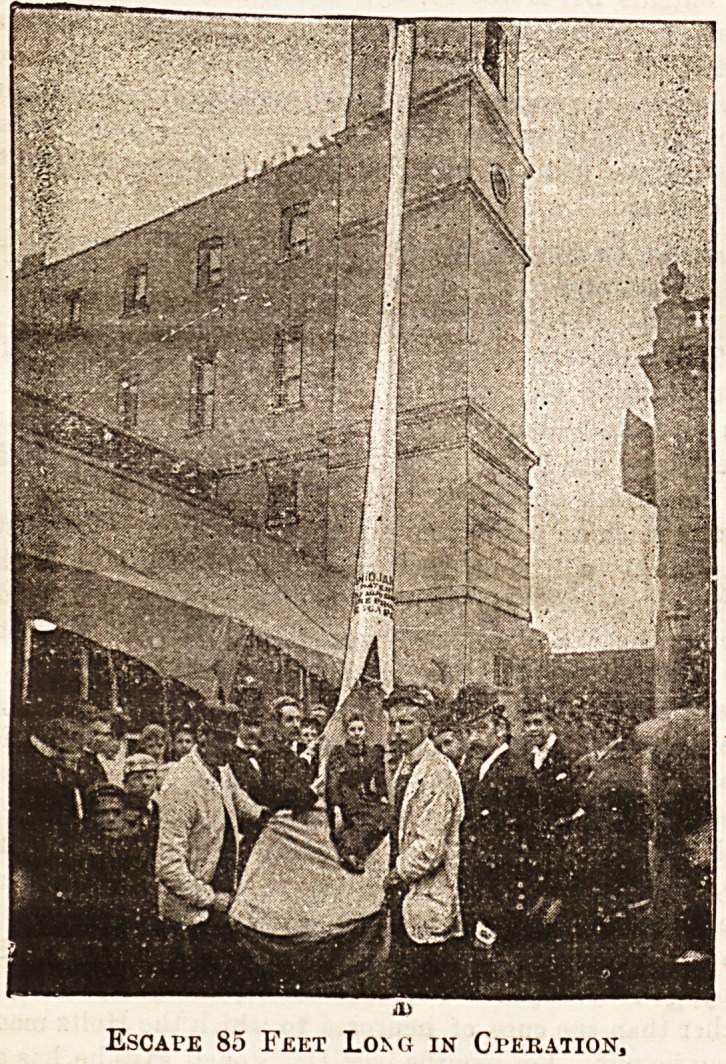# New Appliances and Things Medical

**Published:** 1898-04-02

**Authors:** 


### NEW APPLIANCES AND THINGS MEDICAL.

[We shall be glad to receive, at our Office, 28 & 29, Southampton Street, Strand, London, W.C., from the manufacturers, specimens of all new preparations and appliances which may be brought out from time to time.]

### THE "ANIDJAH" FIRE ESCAPE.

(258, High Holborn, London, W.C.)

This is a most practical and efficient escape. Unless one has had personal experience of the suffocating effect of smoke it is difficult to realise how quickly a staircase may become abso-

Escape 85 Feet Long in Operation.

lutely blocked. The fact is that when a fire commences in the lower part of a house, especially in the case of lofty buildings such as we see in towns, the only chance of safety lies in escape from the windows. We have great pleasure then in drawing attention to the "Anidjah" fire escape, by which this mode of exit is made possible. We have seen it in action, and believe it to be most effectual, and it is so simple that a girl or even an invalid can work it unaided. It consists of a strong canvas tube, sufficiently large to enable a person easily to slide down it. The upper opening of the tube is fixed to an iron framework, which is permanently fastened by hinges to the woodwork of the window. When not wanted it lies rolled up in a box-ottoman or hidden behind a light dressing-table. In case of fire the bottom window sash is raised, the tube is thrown out, and the iron frame at the top of the tube at once catches the sides of the window frame, so that the whole of the window opening is filled by the wide mouth of the tube. This is a matter of great importance. It requires great nerve to step out of a window when one sees the depth below. But with the "Anidjah" escape one sees nothing but what looks like the inside of a canvas tent with a rope hanging in it. The look out into the street is hidden altogether. One takes hold of the rope, steps over the window sill into the tube, and away one goes. The by-standers below having caught the bottom of the tube and drawn it a dozen or twenty feet from the house, hold it firmly in position. When this is done the momentum is taken away by the curve. But even if no one is holding at the bottom a safe descent can be made by aid of the rope. The canvas is treated by chemicals, by which it is rendered "non-inflammable." It is an admirable invention, and among its best features are the rapidity with which a perfect stream of people may be brought down, the ease with which it can be put in operation, and the fact that no one need stick fast from fright and so obstruct its use. When the tube is properly held at the bottom children and invalids can be sent down with great facility and safety. If we had a broken thigh and a long splint on we had a great deal rather be dropped down a chute than carried down a ladder; indeed, it is difficult to imagine a more useful form of fire escape for hospitals and institutions whose inmates are more or less helpless. Like everything else, it should be now and then inspected to see that it is in good order.

### ARMOUR'S EXTRACT OF BEEF.

(Armour and Co., 59, Tooley Street, S.E.)

Armour's Extract of Beef, sent to us, is a preparation of beef in a highly concentrated form. Examination of the sample showed 67 per cent. of meat extractives, including a small proportion of albumen, 20·9 per cent. mineral constituents, and only 12·1 per cent. of water. It was of a good pale brown colour, free from all indications of having been over-heated or burnt, and in every respect an exceedingly well made extract of beef.

### THE ALLENBURY TOILET SOAP.

(Messrs. Allen and Hanburys, London.)

We have received samples of this new toilet soap, and as we have found to be the case with the various medicated soaps supplied by the same firm, it appears to be absolutely pure and free from caustic alkalies, irritating perfumes, and poisonous pigments. It is an exceedingly pleasant soap for general use, and owing to its careful preparation and freedom from excess of water it will be found remarkably economical.

## Figures and Tables

**Figure f1:**